# Structural, Microstructural, and Metabolic Alterations in Primary Progressive Aphasia Variants

**DOI:** 10.3389/fneur.2018.00766

**Published:** 2018-09-18

**Authors:** Alexandre Routier, Marie-Odile Habert, Anne Bertrand, Aurélie Kas, Martina Sundqvist, Justine Mertz, Pierre-Maxime David, Hugo Bertin, Serge Belliard, Florence Pasquier, Karim Bennys, Olivier Martinaud, Frédérique Etcharry-Bouyx, Olivier Moreaud, Olivier Godefroy, Jérémie Pariente, Michèle Puel, Philippe Couratier, Claire Boutoleau-Bretonnière, Bernard Laurent, Raphaëlla Migliaccio, Bruno Dubois, Olivier Colliot, Marc Teichmann

**Affiliations:** ^1^Institut du Cerveau et de la Moelle épinière, ICM, Inserm U 1127, CNRS UMR 7225, Sorbonne Université, FrontLab, Paris, France; ^2^Inria, Aramis Project-Team, Paris, France; ^3^Laboratoire d'Imagerie Biomédicale, Sorbonne Université, Inserm U 1146, CNRS UMR, Paris, France; ^4^AP-HP, Hôpital Pitié-Salpêtrière, Department of Nuclear Medicine, Paris, France; ^5^Centre Acquisition et Traitement des Images, Paris, France; ^6^Institut du Cerveau et de la Moelle épinière, ICM, Inserm U 1127, CNRS UMR 7225, Sorbonne Université, AP-HP, Paris, France; ^7^AP-HP, Hôpital Saint Antoine, Department of Radiology, Paris, France; ^8^Department of Nuclear Medicine, European Hospital Georges Pompidou, Paris, France; ^9^Normandie University, UNICAEN, EPHE, INSERM, U1077, Neuropsychologie et Imagerie de la Mémoire Humaine, Caen, France; ^10^Department of Neurology, Memory Research and Resource Center for Alzheimer's Disease, University Hospital Pontchaillou, Rennes, France; ^11^Department of Neurology, University Hospital of Lille, Lille, France; ^12^Department of Neurology, Memory Research and Resource Center for Alzheimer's Disease, University Hospital of Montpellier, Montpellier, France; ^13^Department of Neurology, University Hospital of Rouen, Rouen, France; ^14^Department of Neurology, Memory Research and Resource Center for Alzheimer's Disease, University Hospital of Angers, Angers, France; ^15^Department of Psychiatry, Neurology and Rehabilitation University Hospital of Grenoble, Memory Research and Resource Center for Alzheimer's Disease, Grenoble, France; ^16^Department of Neurology and Laboratory of Functional Neurosciences (EA 4559), University Hospital of Amiens, Amiens, France; ^17^CHU Toulouse, Neurology Department, Toulouse, France; ^18^INSERM/UPS, UMR 1214—ToNIC, Toulouse NeuroImaging Center, University of Toulouse III, Toulouse, France; ^19^Department of Neurology, University Hospital of Limoges, Limoges, France; ^20^Department of Neurology, University Hospital of Nantes, Nantes, France; ^21^Department of Neurology, University Hospital of Saint-Etienne, Saint-Etienne, France; ^22^Department of Neurology, Institute for Memory and Alzheimer's Disease, Pitié-Salpêtrière Hospital, AP-HP, Paris, France; ^23^National Reference Center for “PPA and rare dementias”, Institute for Memory and Alzheimer's Disease, AP-HP, Paris, France; ^24^Institut du Cerveau et de la Moelle épinière, ICM, Inserm U 1127, CNRS UMR 7225, Sorbonne Université, Paris, France; ^25^AP-HP, Departments of Neuroradiology and Neurology, Hôpital de la Pitié-Salpêtrière, Paris, France

**Keywords:** primary progressive aphasias, cortical thickness, cortical metabolism, tracts, MRI, PET

## Abstract

Neuroimaging studies have described the brain alterations in primary progressive aphasia (PPA) variants (semantic, logopenic, nonfluent/agrammatic). However, few studies combined T1, FDG-PET, and diffusion MRI techniques to study atrophy, hypometabolism, and tract alterations across the three PPA main variants. We therefore explored a large early-stage cohort of semantic, logopenic and nonfluent/agrammatic variants (*N* = 86) and of 23 matched healthy controls with anatomical MRI (cortical thickness), FDG PET (metabolism) and diffusion MRI (white matter tracts analyses), aiming at identifying cortical and sub-cortical brain alterations, and confronting these alterations across imaging modalities and aphasia variants. In the semantic variant, there was cortical thinning and hypometabolism in anterior temporal cortices, with left-hemisphere predominance, extending toward posterior temporal regions, and affecting tracts projecting to the anterior temporal lobes (inferior longitudinal fasciculus, uncinate fasciculus) and tracts projecting to or running nearby posterior temporal cortices: (superior longitudinal fasciculus, inferior frontal-occipital fasciculus). In the logopenic variant metabolic alterations were more extensive than atrophy affecting mainly the left temporal-parietal junction and extending toward more anterior temporal cortices. Metabolic and tract data were coherent given the alterations of the left superior and inferior longitudinal fasciculus and the left inferior frontal-occipital fasciculus. In the nonfluent/agrammatic variant cortical thinning and hypometabolism were located in the left frontal cortex but Broca's area was only affected on metabolic measures. Metabolic and tract alterations were coherent as reflected by damage to the left uncinate fasciculus connecting with Broca's area. Our findings provide a full-blown statistically robust picture of brain alterations in early-stage variants of primary progressive aphasia which has implications for diagnosis, classification and future therapeutic strategies. They demonstrate that in logopenic and semantic variants patterns of brain damage display a non-negligible overlap in temporal regions whereas they are substantially distinct in the nonfluent/agrammatic variant (frontal regions). These results also indicate that frontal networks (combinatorial syntax/phonology) and temporal networks (lexical/semantic representations) constitute distinct anatomo-functional entities with differential vulnerability to degenerative processes in aphasia variants. Finally, the identification of the specific damage patterns could open an avenue for trans-cranial stimulation approaches by indicating the appropriate target-entry into the damaged language system.

## Introduction

Primary Progressive Aphasia (PPA) is a group of neurodegenerative diseases affecting language abilities. PPA have been classified intro three main variants (1): the semantic variant (sv-PPA) characterized by the impairment of the representations of word meanings, the logopenic variant (lv-PPA) surfacing with lexical disorder and a decrease of verbal short-term memory, and the non-fluent/agrammatic variant (nfv-PPA) characterized by phonological/phonetic and syntactic disorders. Several neuroimaging studies have explored the brain alterations in the three PPA variants. Cortical atrophy in sv-PPA is located in anterior temporal lobes with left predominance (2–5), and metabolic alterations of the cortex on FDG-PET usually overlap with these temporal regions (2, 6). White matter damage involves the uncinate fasciculus and the inferior longitudinal fasciculus bilaterally (7, 8). One study also found alterations of the left arcuate fasciculus and the left inferior fronto-occipital fasciculus (9). Lv-PPA is associated with cortical atrophy of the left temporo-parietal junction (3) extending in some studies to more anterior temporal regions (10–13). Hypometabolism is observed in the left temporo-parietal junction, and in left inferior, middle, and superior-posterior temporal cortices (10), which, in some studies, can extend to left prefrontal regions and to the right hemisphere (13–15). White matter alterations involve the left arcuate fasciculus/superior longitudinal fasciculus (7, 8) and the left inferior longitudinal fasciculus (9). Nfv-PPA patients exhibit atrophy in left frontal regions although specific anatomical areas vary from study to study (5, 16, 17). Hypometabolism is found in the inferior frontal gyrus, the middle frontal gyrus and in the supplementary motor area (10, 18). White matter alterations involve the left superior longitudinal fasciculus (7). Involvement of the left uncinate fasciculus (8), the frontal aslant tract (19) or the left inferior frontal-occipital fasciculus (9) has also been described.

Despite the growing body of literature on brain damage in PPA only few studies combined MRI-T1, FDG-PET and MRI-diffusion-weighted-imaging to assess atrophy patterns, metabolic profiles and tract alterations in the three PPA variants. There is currently only one multimodal study on sv-PPA (2), one on lv-PPA (13), and one on nfv-PPA (20) but no investigation has directly compared the three PPA variants. In addition, previous studies often involved small sample sizes (2, 6, 9, 18, 21, 22), uncorrected statistics (18, 23) or cluster-level corrections (13) which are known to result in inflated false positives (24, 25). Finally, several investigations explored PPA patients at advanced disease stages in terms of disease duration which can lead to mixed and non-pure PPA types (7, 22, 26). Thus, a comprehensive multimodal imaging investigation on a large PPA cohort including the three main variants is needed to provide a reliable and full-blown picture of brain damage. The present investigation addressed these issues studying structural (cortical thickness), microstructural (tract parameters) and metabolic alterations in a large population of early-stage patients of the three PPA variants using structural MRI, diffusion-weighted imaging (DWI) and FDG-PET imaging, while applying a rigorously corrected statistical approach.

## Methods

### Participants

Participants were consecutively recruited and included within the French multicenter investigation on primary progressive aphasias (“PHRC-CAPP”). The PPA variant diagnoses were established by expert neurologists following the current international diagnostic criteria (1). PPA variant diagnosis was exclusively based on language disorders, which allowed for characterizing, diagnosing and distinguishing the three main variants. The cognitive and language data of all PPA cases were revised and categorized by the investigators of the 12 centers of the “PHRC-CAPP” investigation to provide a precise and reliable PPA variant diagnosis, based on the international consensus criteria (1). In addition, all the data and the PPA variant classifications were subsequently checked by the principal investigator/coordinator of the “PHRC-CAPP” (M.T.).

One hundred and fourteen participants were included (47 sv-PPA, 28 lv-PPA, 16 nfv-PPA, and 23 healthy controls). PPA patients were at an early stage of the disease as reflected by aphasia severity scores ≥ 3 (normal = 5) in the Boston Diagnostic Aphasia Examination (27). Patients did not present any neurological/psychiatric disease other than PPA. All participants were native French speakers. The French multicenter investigation “PHRC-CAPP” from which the patients were recruited is an investigation promoted, funded and monitored by the “Assistance Publique des Hôpitaux de Paris” (AP-HP). Accordingly, and in line with French legislation, the investigation was therefore approved by an ethics committee of Paris and informed written consent was obtained from the participants.

### Image acquisition

Imaging was conducted at 12 sites across France. Imaging centers belong to the harmonized national network of the *Centre d'Acquisition et de Traitement d'Images* (CATI) (http://cati-neuroimaging.com/) (28, 29). MRI and FDG-PET sequences were harmonized by the CATI in order to minimize differences between centers. The CATI performs onsite visits for the setup of imaging protocols and regular follow-up. Systematic quality checks of acquired images were performed by the CATI using a dedicated software program with quantitative and qualitative indices, which allowed checking for (1) protocol consistency, (2) presence and localization of artifacts, and (3) overall image quality.

T1-weighted images were acquired with a 3D gradient echo sequence (240 × 256 acquired matrix; voxel size = 1.0 × 1.0 × 1.0 mm^3^; inversion time = 900 ms; repetition time 2,300 ms; echo time = 2.98 ms; flip angle = 9°). Diffusion-weighted images (DWI) were acquired using an echo-planar imaging sequence (EPI) (128 × 128 acquired matrix, voxel size: 2.0 × 2.0 × 2.0 mm^3^). Seventy separate images were extracted from each DWI scan: 10 T2-weighted images with no dedicated diffusion sensitization (b0 images) and 60 diffusion-weighted images (b = 1500 s/mm^2^). A fieldmap image was acquired to correct for geometrical distortions induced by the EPI sequence.

FDG-PET scans were obtained 30 min after injection of 2 MBq/kg of 2-deoxy-2-(18F)fluoro-D-glucose (with a minimum dose of 125 MBq and a maximum dose of 250 MBq). PET acquisitions consisted of 3 × 5 min frames. Images were reconstructed using a conventional 3D iterative algorithm, with a post-reconstruction filter in a 128^*^128 matrix. Acquisition parameters were harmonized for 12 different scanners. Voxel size range from 2 to 3.27 mm. Attenuation, scatter and random coincidence corrections were integrated in the reconstruction. Algorithms with spread function modeling were discarded, even if available. Finally, frames were realigned, averaged and quality-checked by the CATI.

One hundred and one participants had both T1 MRI and FDG-PET that passed the quality control procedure (41 sv-PPA, 26 lv-PPA, 12 nfv-PPA patients, and 22 healthy controls). This population formed the T1-PET cohort for which demographical information is summarized in Table 1. Seventy-seven participants had both T1 and diffusion MRI of sufficient quality (32 sv-PPA, 19 lv-PPA, 6 nfv-PPA patients, and 18 healthy controls). This population formed the T1-DWI cohort for which demographical information is summarized in Table 1. Age, gender, years of education, and disease duration did not differ between the groups (univariate ANOVAs using *post-hoc* analysis with Tukey's test for continuous variables, Chi-square test for categorical variables). In summary, a total number of 86 patients (45 sv-PPA, 28 lv-PPA, 13 nfv-PPA) and of 23 healthy controls was included in the study. The inclusion centers of the participants and their recruitment volume is summarized in the Supplementary Table 1.

**Table 1:** Demographic information and standard cognitive/language assessment of the T1-PET and the T1-DWI cohorts.

**Demographics**	**Controls**	**sv-PPA**	**lv-PPA**	**nfv-PPA**	
**T1-PET COHORT**					
Number of subjects	22	41	26	12	
Gender (male/female)	11F/11M	21F/20M	11F/15M	7F/5M	
Handedness (right/left)	1L/21R	1L/40R	4L/22R	1L/11R	
Symptom duration (years)	/////	2.63 ± 1.72	2.58 ± 1.69	2.50 ± 1.89	
Age (years)	65.86 ± 7.61	66.41 ± 6.62	68.54 ± 5.35	68.42 ± 5.51	
Years of education	13.00 ± 2.11	11.98 ± 4.96	12.85 ± 4.43	11.58 ± 3.68	
**Cognitive/language assessment**					**Normal threshold**
MMSE	27.55 ± 1.41	24.73 ± 2.52*	24.88 ± 3.61	24.67 ± 2.98	≥27
FAB	17.00 ± 0.85	14.78 ± 1.79	13.46 ± 2.78^*^	11.17 ± 4.91^*^^, sv^	≥16
MDRS	141.23 ± 2.50	117.66 ± 12.10	120.58 ± 15.08	111.58 ± 17.75	≥137
**LANGUAGE TESTS**					
Severity of aphasia (BDAE)	5.00 ± 0.00	3.66 ± 0.47^*^	3.58 ± 0.57^*^	3.33 ± 0.47^*^	> 4
Single-word comprehension (BDAE)	71.68 ± 0.55	61.37 ± 7.89^*^^, lv, nfv^	69.50 ± 2.96	68.58 ± 5.31	≥68
Sentence repetition (BDAE)	15.86 ± 0.34	14.66 ± 1.76	10.92 ± 4.16^*^^, sv^	14.25 ± 1.69^*^	≥14
DO80–Global (Picture naming)	79.68 ± 0.55	43.46 ± 19.89^*^^, lv, nfv^	65.46 ± 14.46	75.25 ± 3.17	≥75
DO80–Number of non-answers	0.05 ± 0.21	17.80 ± 14.67^*^^, nfv^	11.46 ± 14.89	3.92 ± 3.20	/////
DO80–Phonemic paraphasias	0.00 ± 0.00	0.22 ± 0.61	0.96 ± 1.53	4.67 ± 1.97^*^	/////
DO80–Semantic paraphasias	0.05 ± 0.21	11.22 ± 9.14^*^^, lv, nfv^	2.42 ± 1.96	0.42 ± 0.49	/////
Letter fluency (“P”/2 min)	25.36 ± 7.06	11.44 ± 5.64^*^	13.27 ± 8.00^*^	8.92 ± 5.28^*^	≥15
Category fluency (“fruits”/2 min)	21.55 ± 5.09	8.17 ± 4.67^*^	9.62 ± 5.21^*^	9.67 ± 5.39^*^	≥15
**T1-DWI COHORT**					
Number of subjects	18	32	19	6	
Gender (male/female)	11F/7M	14F/18M	9F/10M	3F/3M	
Handedness (right/left)	0L/18R	1L/31R	4L/15R	1L/5R	
Symptom duration (years)	/////	2.56 ± 1.60	2.26 ± 1.48	1.83 ± 1.07	
Age (years)	64.67 ± 6.18	66.91 ± 6.82	68.53 ± 6.53	70.50 ± 5.62	
Years of education	13.06 ± 2.15	12.62 ± 5.15	14.42 ± 4.18	12.67 ± 2.43	
**Cognitive/language assessment**					**Normal threshold**
MMSE	27.56 ± 1.34	24.81 ± 2.58^*^	25.21 ± 2.89	25.67 ± 3.09	≥27
FAB	17.06 ± 0.85	14.66 ± 1.93	13.58 ± 2.62^*^	13.00 ± 3.65	≥16
MDRS	141.28 ± 2.68	119.38 ± 11.69	121.53 ± 12.37	121.33 ± 20.19	≥137
**LANGUAGE TESTS**					
Severity of aphasia (BDAE)	5.00 ± 0.00	3.69 ± 0.46^*^	3.68 ± 0.46^*^	3.50 ± 0.50^*^	≥4
Single-word comprehension (BDAE)	71.67 ± 0.58	61.28 ± 7.88^*^^, lv, nfv^	70.16 ± 2.74	70.83 ± 0.90	≥68
Sentence repetition (BDAE)	15.89 ± 0.31	14.78 ± 1.76	11.53 ± 3.90^*^^, sv^	13.90 ± 1.38^*^	≥14
DO80–Global (Picture naming)	79.61 ± 0.59	42.25 ± 19.14^*^^, lv, nfv^	65.74 ± 13.85	76.00 ± 3.61	≥75
DO80–Number of non-answers	0.06 ± 0.23	18.50 ± 14.30^*^^, nfv^	11.47 ± 14.36	3.17 ± 3.62	/////
DO80–Phonemic paraphasias	0.00 ± 0.00	0.25 ± 0.66	0.63 ± 1.27	5.17 ± 2.11^*^^, sv^	/////
DO80–Semantic paraphasias	0.06 ± 0.23	12.12 ± 9.90^*^^, lv, nfv^	2.58 ± 2.09	0.00 ± 0.00	/////
Letter fluency (“P”/2 min)	24.78 ± 6.71	11.62 ± 5.76^*^	12.32 ± 5.55^*^	11.17 ± 4.74^*^	≥15
Category fluency (“fruits”/2 min)	20.78 ± 4.96	8.00 ± 5.07^*^	10.05 ± 5.85^*^	12.00 ± 6.43^*^	≥15

Values are presented as means ± standard deviations.

*F, female; M, male; L, left-handed; R, right-handed. MMSE, Mini-Mental State Examination; FAB, Frontal Assessment Battery; MDRS, Mattis Dementia Rating Scale; BDAE, Boston Diagnostic Aphasia Evaluation; DO80, picture naming test. The “Normal threshold” column shows available normative scores of the used standard tests, corresponding to the age and to the years of education for each of the four participant groups. Asterisks denote statistically significant differences with respect healthy controls (P < 0.05). Superscript letters denote statistically significant differences relative to the ^sv^semantic, ^lv^logopenic and ^nfv^non-fluent variants (P < 0.05). Univariate ANOVAs using post-hoc analysis with Tukey's test were used to compare the different groups*.

### Cognitive/language assessment

The general cognitive assessment included the Mini Mental State Examination (30), the Mattis Dementia Rating Scale (31), and the Frontal Assessment Battery (32). The language assessment was based on the Boston Diagnostic Aphasia Evaluation (27) including an evaluation of the severity of aphasia, taking into account spontaneous speech and the description of the “cookies theft picture,” a sentence repetition task, and a single-word comprehension task requiring pointing to pictures upon auditory word presentation. We also applied a picture naming test (DO80) (33), and a verbal fluency test comprising phonemic and category fluency (34). We assessed differences between the four groups using univariate ANOVAs and differences for each possible pair of groups using *post-hoc* analysis with Tukey's test. Significance levels were set at *P* < 0.05. Cognitive/language scores for the T1-PET cohort and the T1-DWI cohort, along with the statistical test results, are presented in Table 1. ANOVA showed significant differences for all scores except the MDRS. The three PPA groups did not differ regarding the severity of aphasia. Impairment for the different scores was consistent with the typical patterns expected for the three PPA variants.

### Structural MRI analyses

Structural T1 MRI data were studied with surface-based cortical thickness analysis using the following procedure, based on FreeSurfer and SurfStat software. T1-weighted images were processed using t1-freesurfer-cross-sectional pipeline of the Clinica (http://www.clinica.run) platform. This pipeline is a wrapper of different tools of the FreeSurfer image analysis software (stable version 5.3; http://surfer.nmr.mgh.harvard.edu) (35). Briefly, the processing pipeline included non-uniformity and intensity correction, skull stripping, gray/white matter segmentation, reconstruction of the cortical surface, cortical thickness estimation, and spatial normalization onto the FreeSurfer surface template (FsAverage). After segmentation, all datasets were checked visually for segmentation errors (errors of GM/WM and GM/CSF boundaries).

Each patient group was compared to the group of healthy controls using surface-based analysis of cortical thickness using the statistics-surfstat command of Clinica. More precisely, a point-wise, vertex-to-vertex model based on the Matlab SurfStat toolbox (http://www.math.mcgill.ca/keith/surfstat/) was used to analyze cortical thickness. The data were smoothed using a Gaussian kernel with a full width at half maximum (FWHM) set to 20 mm. Statistical analysis was performed using general linear model with age and sex as covariates. Statistics were corrected for multiple comparisons using the random field theory for non-isotropic images (36) with family-wise error correction at the vertex level. A statistical threshold of *P* < 0.05 corrected for multiple comparisons was applied.

### Diffusion MRI analyses

Diffusion MRI data were studied using region-of interest analysis of diffusion tensor imaging (DTI) metrics, using the following procedure that combines tools from FSL and ANTs software. Preprocessing of diffusion data was performed with Clinica. First, we aligned for each subject all raw DWI volumes to the average b0 image (DWI volume with no diffusion sensitization) with 6 degrees of freedom to correct for head motion, and the diffusion weighted directions were appropriately updated as recommended by Leemans and Jones (37). A registration with 12 degrees of freedom was used to correct for eddy current distortions. These registrations were done using the FSL flirt tool (www.fmrib.ox.ac.uk/fsl). To correct for EPI-induced susceptibility artifacts, the fieldmap image was used as proposed by Jezzard and Balaban (38) with the FSL prelude/fugue tools. Finally, the DWIs were corrected for non-uniform intensity using ANTs N4 bias correction algorithm (39). A single multiplicative bias field from the averaged b0 image was estimated, as suggested by Jeurissen et al. (40).

A diffusion tensor model was fitted at each voxel to calculate Fractional Anisotropy (FA) and Mean Diffusivity (MD) maps. We then assessed the integrity of a set of anatomical white matter tracts defined in the *JHU* white-matter tractography atlas (41). This atlas contains 20 white matter tract labels that were identified probabilistically by averaging the results of deterministic tractography run on 28 subjects. Several thresholds of these probabilistic tracts are proposed (0, 25, 50%). After visual inspection, the 25% threshold was selected which was neither too noisy (compared to the 0% threshold) nor too selective (compared to the 50% threshold). For each subject, the FA map of the subject was registered onto the FA map of the *JHU* atlas template with the ANTs SyN algorithm (42). Then, the estimated non-linear deformation was applied to the MD maps so that both the FA and MD maps of each subject were put into correspondence with the atlas. The implementation of these different steps is available in the dwi-processing-dti pipeline of Clinica.

Differences in tract integrity (FA and MD measures in each tract) between each PPA variant and healthy controls were assessed using general linear model with age and sex as covariates. Statistics were corrected for multiple comparisons using the Bonferroni correction and a statistical threshold of *P* < 0.05 corrected for multiple comparisons was applied.

### FDG-PET analyses

A voxel-based analysis of PET data from the three PPA variants and the healthy controls was performed using a pipeline developed by the CATI and SPM software. PET volumes were co-registered to their corresponding MRI volumes. MRI volumes were segmented into gray matter, white matter, and cerebrospinal fluid probability maps using SPM12 (http://www.fil.ion.ucl.ac.uk/spm). We then applied a partial volume effect correction algorithm that performs a region-based voxel-wise (RBV) correction of the entire image (43), using the anatomical parcellation of MRI scans and an accurate measure of the point spread function of the PET scanners. MRI volumes were spatially normalized to MNI space. PET co-registered images were spatially normalized applying the transformation parameters of MRI normalization. The PET images in the MNI space were then intensity normalized according to a reference region to obtain a standardized uptake value ratio (SUVR) map. The reference region was the pons, which is a known region to be preserved in Alzheimer's Disease and shown to adequately reflect inter-individual variability (44). In particular, we used the pons region obtained after erosion from Pickatlas (http://fmri.wfubmc.edu/software/pickatlas).

Finally, we performed a voxel-based analysis with SPM12. The normalized data were smoothed with an isotropic Gaussian kernel of 8 mm. Statistical analysis was performed using general linear model with age and sex as covariates. An explicit FDG-PET mask was created (45) by merging the different regions of the AAL2 atlas (46). Statistics were corrected for multiple comparisons with family-wise error (FWE) correction at the peak level. A statistical threshold of *P* < 0.05 FWE corrected was applied and significant clusters containing more than 100 voxels were taken into consideration.

## Results

### Structural alterations of the cortex

**Figure 1:**
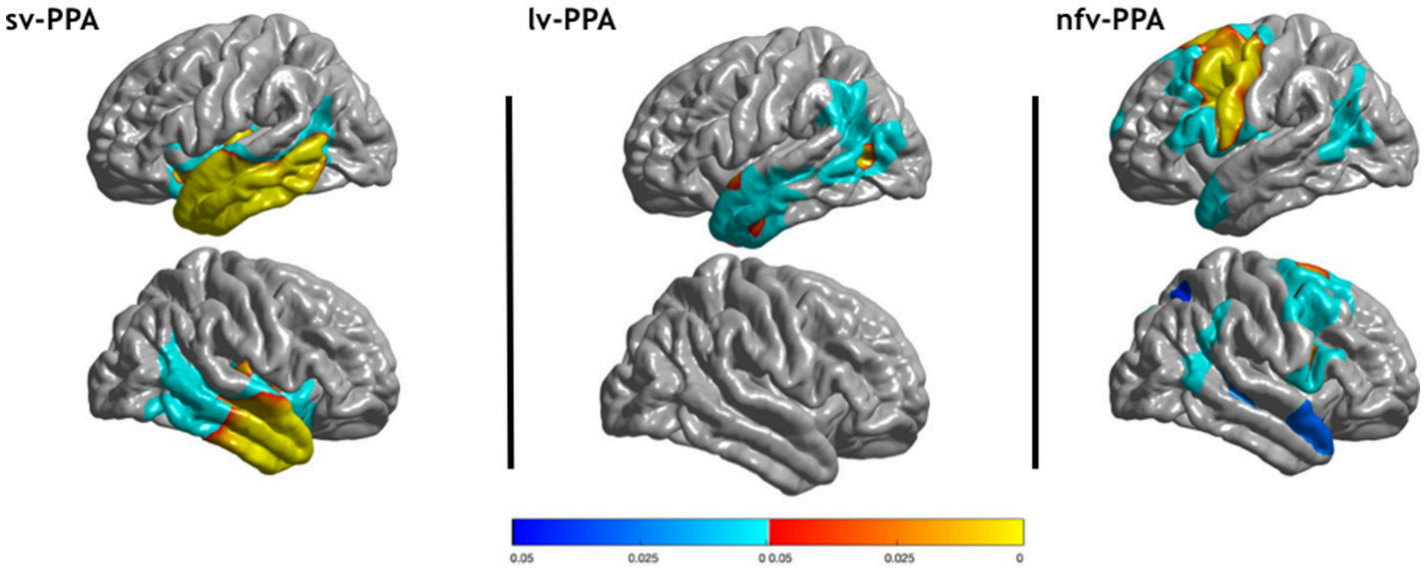
Areas of significantly reduced cortical thickness in sv-PPA, lv-PPA and nfv-PPA, compared to healthy controls. Corrected *p*-values at the vertex-level and the cluster-level are displayed with red/yellow and blue colors, respectively.

Results from group comparisons for cortical thickness with vertex-level correction are illustrated in Figure 1 (red/yellow colormap). Sv-PPA showed reduction of cortical thickness in the anterior temporal lobes predominating in the left hemisphere. Cortical thickness alterations extended toward left posterior temporal regions. Lv-PPA demonstrated small clusters of thickness reduction in the left middle posterior temporal gyrus and the left anterior temporal cortex. In nfv-PPA cortical thinning was located in the left premotor cortex, the left supplementary motor area and the left primary motor area.

We also assessed a cluster-level correction which is known to be less conservative than vertex-level correction. A statistical threshold of *P* < 0.001 was first applied and a threshold of *P* < 0.05 corrected for multiple comparisons was then applied at the cluster level. The results are displayed in Figure 1 (blue colormap). With cluster-level correction, cortical thickness reductions were more widespread in the three PPA variants: lv-PPA showed two clusters including the left temporal-parietal junction and the left anterior temporal cortex, sv-PPA exhibited clusters including large regions of the temporal lobes and some small portions the inferior frontal gyrus, nfv-PPA showed clusters including frontal regions and some portions of the temporal and parietal lobes.

### Metabolic alterations of the cortex

**Figure 2:**
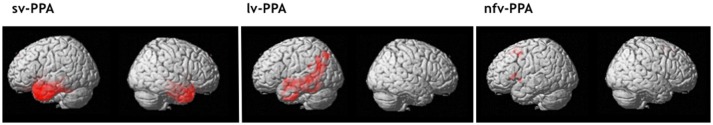
Areas of significant hypometabolism in sv-PPA, lv-PPA and nfv-PPA, compared to healthy controls. The maps display *p*-values, corrected for multiple comparisons using peak-level FWE correction (*P* < 0.05).

Results from group comparisons for FDG-PET metabolism are illustrated in Figure 2. Sv-PPA patients showed bilateral left-predominant hypometabolism in the anterior temporal cortices, extending to the left cingulate and toward middle/posterior temporal regions. Lv-PPA patients showed alterations in the left temporal-parietal junction and left inferior, middle, and superior temporal regions extending toward anterior temporal cortices. Nfv-PPA patients demonstrated hypometabolism in regions of the left inferior frontal gyrus (including the pars opercularis/triangularis of Broca's area [Brodmann area 44/45]), of the middle frontal gyrus and the supplementary motor area. Results with less severe cluster-level corrections are shown in the Supplementary Figure 1.

### Microstructural tract alterations

**Figure 3:**
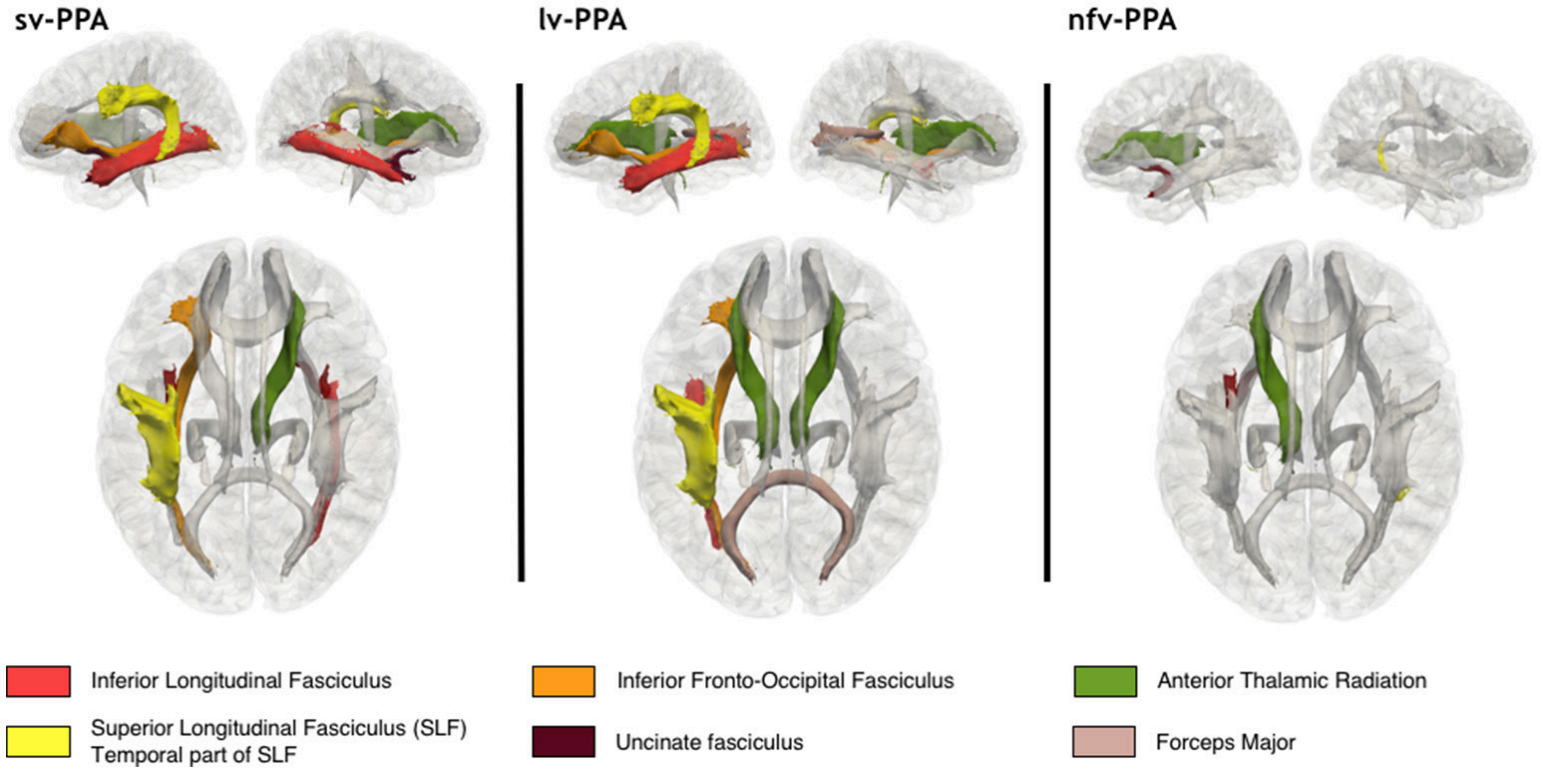
Alterations of white matter tracts in sv-PPA, lv-PPA, and nfv-PPA, compared to healthy controls. Colored tracts are altered for FA and/or MD measures at Bonferroni corrected *p*-values < 0.05.

Results from group comparisons for microstructural white matter tract alterations are illustrated in Figure 3, and *p*-values for MD and FA abnormalities are presented in Table 2. Sv-PPA showed bilateral alterations in tracts making connections with the anterior temporal cortex: the left and right inferior longitudinal fasciculus (ILF) for MD, and the left and right uncinate fasciculus (UF) for FA and MD. We also found alterations in tracts connecting or passing close to posterior temporal regions: the left superior longitudinal fasciculus (SLF) for FA and the left inferior frontal-occipital fasciculus (IFOF) for FA. In addition, the right anterior thalamic radiations (ATR) were altered for FA measures. Lv-PPA patients had alterations of the left SLF for MD and FA, the left IFOF for FA and MD, and the left ILF for MD. In addition, the forceps major was altered for FA, and the ATR were bilaterally altered for MD measures. Nfv-PPA patients demonstrated alterations for MD of the left UF, the left ATR and the temporal part of the right SLF.

**Table 2:** Alterations of white matter tracts for each DTI metric (FA and MD) in sv-PPA, lv-PPA and nfv-PPA compared to healthy controls.

**Tracts**	**HC**	**sv-PPA**	**lv-PPA**	**nfv-PPA**
**RESULTS FOR FA**				
L-ILF	0.372	0.348 (*p* = 1, T = −1.53)	0.358 (*p* = 1, T = −1.00)	0.357 (*p* = 1, T = −0.62)
R-ILF	0.402	0.384 (*p* = 1, T = −1.16)	0.392 (*p* = 1, T = −0.43)	0.396 (*p* = 1, T = −0.14)
L-SLF	0.377	0.**340 (*****p*** = **0.0085, T** = −**3.81)**	**0.338 (*****p*** = **0.0225, T** = −**3.59)**	0.343 (*p* = 0.81, T = −2.21)
R-SLF	0.405	0.371 (*p* = 0.19, T = −2.72)	0.370 (*p* = 0.143, T = −2.89)	0.376 (*p* = 1, T = −1.72)
L-TP-SLF	0.470	0.424 (*p* = 0.12, T = −2.89)	**0.398 (*****p*** = **0.0273, T** = −**3.52)**	0.455 (*p* = 1, T = −0.28)
R-TP-SLF	0.478	0.438 (*p* = 0.51, T = −2.32)	0.463 (*p* = 1, T = 0.19)	0.492 (*p* = 1, T = 1.93)
L-UF	0.363	**0.283 (*****p*** = **8.96e**−**05, T** = −**5.20)**	0.325 (*p* = 0.36, T = −2.51)	0.321 (*p* = 0.92, T = −2.15)
R-UF	0.391	**0.318 (*****p*** = **0.0060, T** = −**3.92)**	0.376 (*p* = 1, T = −0.78)	0.354 (*p* = 1, T = −1.74)
L-IFOF	0.435	**0.384 (*****p*** = **0.0182, T** = −**3.56)**	**0.392 (*****p*** = **0.0103, T** = −**3.87)**	0.424 (*p* = 1, T = −0.50)
R-IFOF	0.437	0.401 (*p* = 0.075, T = −3.07)	0.407 (*p* = 0.302, T = −2.58)	0.424 (*p* = 1, T = −0.59)
L-ATR	0.375	0.340 (*p* = 0.35, T = −2.47)	0.344 (*p* = 0.0955, T = −3.05)	0.353 (*p* = 1, T = −1.26)
R-ATR	0.356	**0.325 (*****p*** = **0.0147, T =** −**3.63)**	0.325 (*p* = 0.156, T = −2.85)	0.316 (*p* = 0.94, T = −2.14)
FM	0.529	0.510 (*p* = 1, T = −0.91)	**0.470 (*****p*** = **0.0244, T** = −**3.56)**	0.478 (*p* = 1, T = −1.66)
Fm	0.374	0.338 (*p* = 0.23, T = −2.65)	0.347 (*p* = 1, T = −2.04)	0.345 (*p* = 1, T = −1.33)
L-CST	0.535	0.5192 (*p* = 1, T = −1.23)	0.523 (*p* = 1, T = −1.45)	0.524 (*p* = 1, T = −0.56)
R-CST	0.548	0.5305 (*p* = 1, T = −1.62)	0.524 (*p* = 0.30, T = −2.57)	0.495 (*p* = 1, T = −2.09)
L-CCG	0.422	0.364 (*p* = 0.14, T = −2.81)	0.354 (*p* = 0.87, T = −2.12)	0.343 (*p* = 1, T = −1.15)
R-CCG	0.360	0.323 (*p* = 0.93, T = −2.06)	0.326 (*p* = 1, T = −1.11)	0.343 (*p* = 1, T = 0.17)
L-CH	0.358	0.338 (*p* = 0.14, T = −0.43)	0.323 (*p* = 0.16, T = −2.84)	0.322 (*p* = 1, T = −1.17)
R-CH	0.370	0.323 (*p* = 0.054, T = −3.18)	0.327 (*p* = 0.33, T = −2.54)	0.316 (*p* = 1, T = −1.64)
**RESULTS FOR MD**				
L-ILF	1.03	**1.26 (*****p*** = **0.0018, T** = **4.30)**	**1.15 (*****p*** = **0.0012, T** = **4.61)**	1.16 (*p* = 0.34, T = 2.62)
R-ILF	1.02	**1.21 (*****p*** = **0.0183, T** = **3.56)**	1.08 (*p* = 1, T = 2.02)	1.09 (*p* = 1, T = 1.62)
L-SLF	1.04	1.09 (*p* = 1, T = 1.11)	**1.11 (*****p*** = **0.0208, T** = **3.61)**	1.16 (*p* = 0.14, T = 3.02)
R-SLF	1.00	1.01 (*p* = 1, T = −0.32)	1.05 (*p* = 0.075, T = 3.14)	1.13 (*p* = 0.68, T = 2.30)
L-TP-SLF	1.06	1.10 (*p* = 1, T = 1.56)	1.13 (*p* = 0.81, T = 2.15)	1.17 (*p* = 1, T = 2.07)
R-TP-SLF	1.05	1.09 (*p* = 1, T = 1.69)	1.09 (*p* = 0.012, T = 2.95)	**1.14 (*****p*** = **0.0259, T** = **3.76)**
L-UF	1.09	**1.54 (*****p*** = **6.08e**−**06, T** = **5.98)**	1.23 (*p* = 0.33, T = 2.54)	**1.30 (*****p*** = **0.0067, T** = **4.34)**
R-UF	1.08	**1.41 (*****p*** = **0.022, T = 3.49)**	1.15 (*p* = 1, T = 1.58)	1.22 (*p* = 0.45, T = 2.49)
L-IFOF	1.13	1.19 (*p* = 1, T = 1.41)	**1.21 (*****p*** = **0.0236, T** = **3.57)**	1.21 (*p* = 0.82, T = 2.20)
R-IFOF	1.08	1.15 (*p* = 0.71, T = 2.19)	1.14 (*p* = 0.705, T = 2.22)	1.14 (*p* = 1, T = 1.87)
L-ATR	1.02	1.20 (*p* = 0.78, T = 2.14)	**1.20 (*****p*** = **0.0043, T** = **4.17)**	**1.32 (*****p*** = **0.0143, T** = **4.01)**
R-ATR	1.04	1.23 (*p* = 0.26, T = 2.60)	**1.21 (*****p*** = **0.0183, T** = **3.66)**	1.33 (*p* = 0.25, T = 2.76)
FM	1.29	1.33 (*p* = 1, T = 0.65)	1.42 (*p* = 0.07, T = 3.16)	1.39 (*p* = 1, T = 1.28)
Fm	1.08	1.08 (*p* = 1, T = −0.45)	1.16 (*p* = 0.409, T = 2.46)	1.21 (*p* = 0.26, T = 2.74)
L-CST	1.16	1.23 (*p* = 1, T = 0.58)	1.22 (*p* = 0.507, T = 2.36)	1.01 (*p* = 1, T = 1.26)
R-CST	1.16	1.15 (*p* = 1, T = −0.34)	1.19 (*p* = 1, T = 1.19)	2.41 (*p* = 1, T = 1.35)
L-CCG	1.06	1.06 (*p* = 1, T = −0.12)	1.09 (*p* = 1, T = 1.66)	1.12 (*p* = 1, T = 2.06)
R-CCG	0.975	0.990 (*p* = 1, T = −0.03)	1.02 (*p* = 0.319, T = 2.56)	1.05 (*p* = 1, T = 1.83)
L-CH	1.03	1.21 (*p* = 1, T = 1.81)	1.17 (*p* = 0.064, T = 3.20)	1.23 (*p* = 0.077, T = 3.29)
R-CH	1.04	1.17 (*p* = 0.82, T = 2.12)	1.07 (*p* = 1, T = 0.52)	1.14 (*p* = 1, 1, T = 1.77)

Mean DTI metric, Bonferroni corrected p-values and T values are displayed. Mean diffusivity values are measured in mm2/s x 10–3.

*L, left; R, right; ILF, Inferior Longitudinal Fasciculus; SLF, Superior Longitudinal Fasciculus; TP-SLF, Temporal Part of the SLF; UF, Uncinate Fasciculus; IFOF, Inferior Fronto-Occipital Fasciculus; ATR, anterior thalamic radiations; CST, Corticospinal Tract; CCG, Cingulum (Cingulate Gyrus); CH, Cingulum (Hippocampus); FM, Forceps Major; Fm, Forceps minor. Significant p-values are indicated in bold*.

### Alterations across imaging modalities

We then aimed at visually comparing the spatial extent of alterations found in the three imaging modalities. To that purpose, we superimposed the areas of significant alterations for the three modalities on the single-subject MNI template (also known as Colin27 template). Mapping of FDG-PET and DWI tracts was straightforward since results are in the MNI space. To map cortical thickness alterations, the areas were transported from the FsAverage template to the single-subject MNI. To this end, we combined different tools from FreeSurfer to transport vertices of cortical alterations (mri_label2label command) and to generate the corresponding volume (mri_label2label and mri_aparc2aseg commands). One should note that some authors have compared imaging modalities through the comparison of corresponding Z-scores (47) or using Cohen's kappa scores (6), but such procedures rely on the computation of single-subject voxel-based statistics which have been shown to result in high false positive rates (25). We therefore used only group-level statistics with well-established and robust procedures.

**Figure 4:**
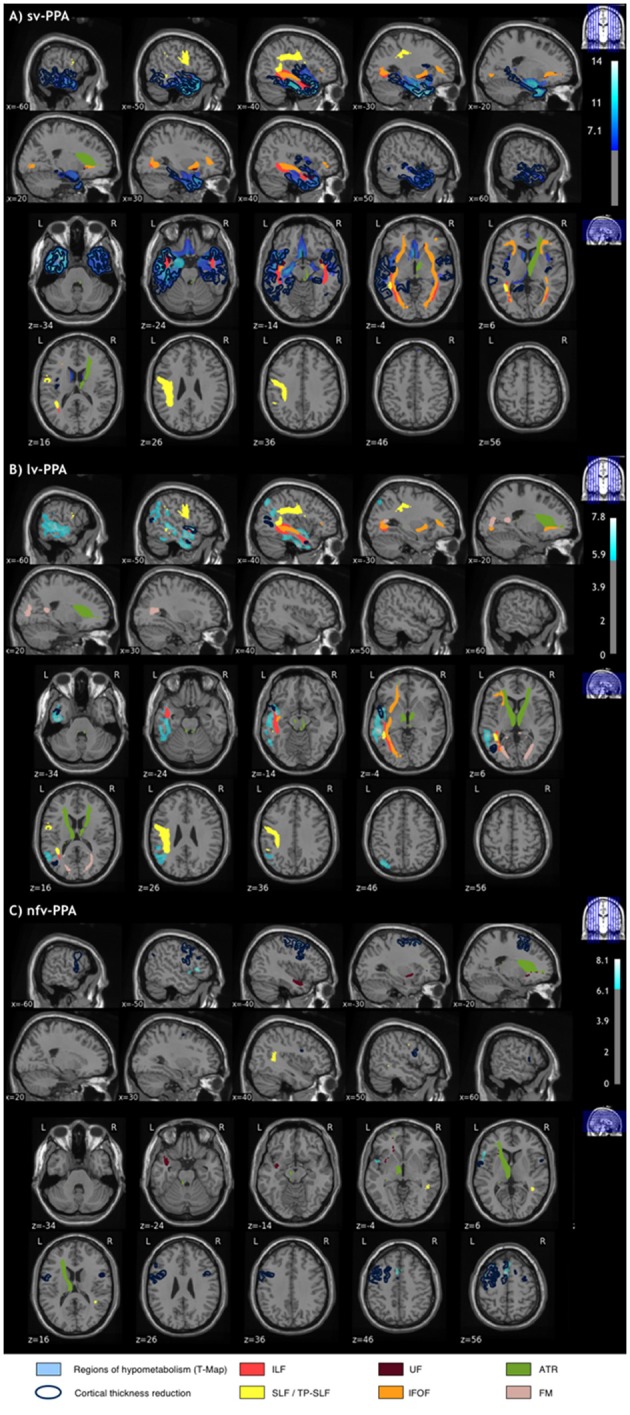
Alterations across the three imaging modalities (T1-cortical thickness, FDG-PET, DWI-tracts) in **(A)** sv-PPA **(B)** lv-PPA **(C)** nfv-PPA patients, compared to healthy controls. Significantly altered areas correspond to a corrected *p*-value of 0.05 for all modalities. The correction for multiple comparisons used FWE at vertex-level for T1, FWE at peak-level for PET, and Bonferroni for DWI.

The obtained visualization for sv-PPA is illustrated in Figure 4A. Alterations are highly coherent for the three modalities. The spatial extent of cortical thickness and metabolic alterations was similar: the anterior temporal lobes with left predominance extending toward middle/posterior temporal regions. Furthermore, FA/MD alterations involved tracts projecting to the hypometabolic and atrophic cortical areas: the ILF and UF projecting to the anterior temporal lobe, the SLF projecting to the posterior temporal cortex, and the IFOF passing close to posterior temporal regions. The right ATR were altered without corresponding areas of atrophy or hypometabolism.

Visualization for the lv-PPA group is presented in Figure 4B. PET alterations were more extensive than cortical thickness reductions when using the peak-level correction for both modalities (extensive cortical thickness alterations were only visible at the cluster-level). In particular, hypometabolism of the left temporal-parietal junction extended toward the left anterior temporal lobe. PET and tract measures displayed coherent alterations, i.e., hypometabolism in the left temporal-parietal junction and alterations of the SLF projecting to posterior temporal regions, and of the IFOF and ILF bordering this region. Finally, the forceps major, the left and right ATR were altered without corresponding to areas of hypometabolism.

Visualization for the nfv-PPA group is displayed in Figure 4C. Cortical thickness and PET alterations were both located in the left frontal cortex but they showed little overlap. Overlap was found in small regions of the primary motor cortex, the premotor cortex, and the supplementary motor area. Alterations of the primary motor and premotor cortex were more extensive on cortical thickness than on metabolic data. On the other hand, cortical alterations of Broca's area were found only on PET. Coherent abnormalities of tract parameters were found in the left UF connecting Broca's area with the anterior temporal cortex. Furthermore, alterations were found in the temporal part of the right SLF which does not connect with atrophic or hypometabolic areas.

### Alterations across PPA variants

**Figure 5:**
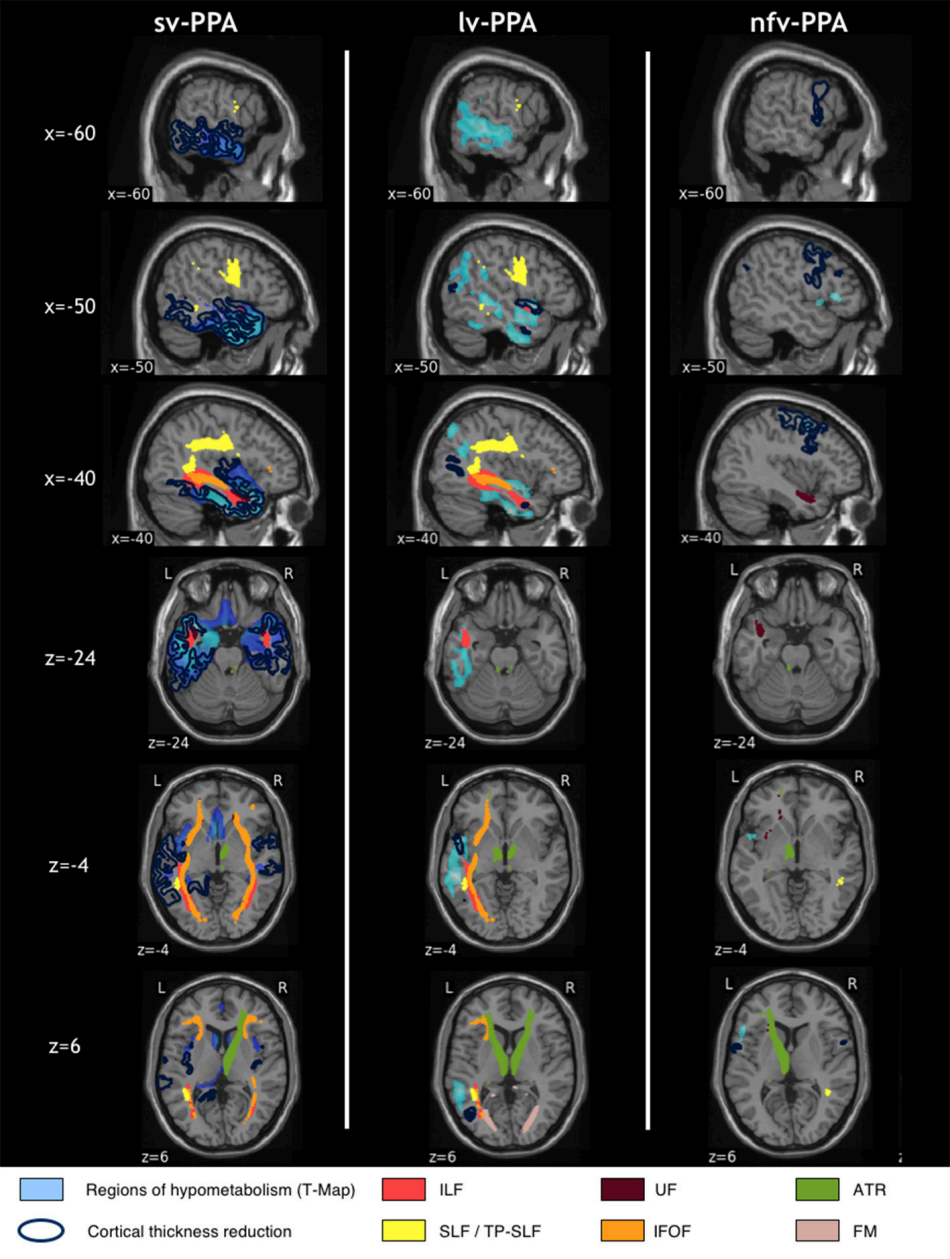
Side-by-side visualization of multimodal alterations in sv-PPA, lv-PPA and nfv-PPA, compared to healthy controls. Significantly altered areas correspond to a corrected *p*-value of 0.05 for all modalities. The correction for multiple comparisons used FWE at the vertex-level for T1, FWE at peak-level for PET and Bonferroni for DWI.

In order to appreciate similarities and differences between the three PPA variants, their corresponding multimodal visualizations were displayed side-by-side (Figure 5). Sv-PPA and lv-PPA patients shared several alterations. Even though PET alterations have different epicenters (the left anterior temporal lobe for sv-PPA and the left temporal-parietal junction for lv-PPA), hypometabolism in lv-PPA extended toward the anterior temporal lobe, and hypometabolism/atrophy in sv-PPA extended to middle/posterior temporal cortices. Moreover, they shared common tract alterations with respect to the SLF, IFOF, and ILF. Sv-PPA and nfv-PPA patients shared no brain alterations except damage to the left UF. Finally, there were no common brain alterations in lv-PPA and nfv-PPA patients.

## Discussion

This multimodal imaging study combined structural MRI, diffusion MRI and FDG-PET in a large cohort of the three early-stage main variants of PPA to identify cortical and sub-cortical brain alterations, and to confront them across imaging modalities and PPA variants. Rigorous statistical procedures allowed for a robust demonstration of commonalities and differences between cortical thickness, metabolic and microstructural tract alterations, and of cerebral damage between PPA variants.

Brain damage predominated in the left hemisphere across imaging modalities and PPA variants. In sv-PPA, alterations were highly coherent for the three imaging modalities showing cortical thinning and hypometabolism in anterior temporal cortices, with left hemisphere predominance, extending toward more posterior left temporal regions, and affecting tracts projecting to the anterior temporal lobes: the ILF and UF (48), and tracts projecting to or passing nearby the posterior temporal cortex: the SLF and the IFOF (48). In lv-PPA, metabolic PET alterations were more extensive than cortical thickness reductions affecting mainly the left temporal-parietal junction and extending toward more anterior left temporal cortices. PET and tract data displayed coherent alterations given the damage to the left SLF, left IFOF and left ILF. In nfv-PPA, both cortical thickness and metabolic PET findings demonstrated alterations in left frontal cortices which were more extensive for cortical thickness reduction. Thickness reduction affected mainly the premotor cortex and motor areas whereas metabolic alterations were primarily found in Broca's area, in addition to motor and premotor regions. Tract alterations were coherent with PET findings as reflected by damage to the left UF which connects Broca's area with the anterior temporal cortex (48, 49). Finally, some additional tracts were altered without corresponding to areas of hypometabolism or atrophy in each PPA variant (ATR, forceps major).

Regarding the analysis across PPA variants, our findings demonstrate that sv-PPA and lv-PPA patients share numerous cortical and tract alterations but they differ by distinct epicenters of cortical damage which is located in left anterior temporal regions in sv-PPA and in posterior temporal and posterior-inferior parietal regions in lv-PPA. Furthermore, damage to the UF is specific to sv-PPA. In contrast, brain damage in nfv-PPA is substantially distinct from sv-PPA and lv-PPA, with the exception of damage to the left UF which is altered in both nfv-PPA and sv-PPA.

Our results are consistent with numerous findings of the literature but they crucially validate, enrich and extend them by providing a comprehensive picture of brain damage applying three-modal imaging with rigorous statistics to a large cohort of the three PPA main variants assessed in early disease stages. In sv-PPA, structural and metabolic alterations of the anterior temporal cortex, implementing semantics (50), is in line with most previous studies [e.g., (2, 3, 6)]. However, the extension of cortical damage toward the left anterior cingulate has not been reported. The involvement of middle/posterior temporal cortices, implementing lexical representations (51–53) has only been reported in smaller scale studies involving more advanced patients (2, 17). On the other hand, we did not find alterations of the orbitofrontal cortex which have been reported by Mesulam et al. (17). Furthermore, findings regarding tract alterations in sv-PPA are inconsistent across the literature which primarily highlighted damage to the ILF and the UF which project to anterior temporal cortices (2, 16, 54) whereas some investigations reported alterations of the SLF and the IFOF which run in the posterior temporal lobe (7, 9). Our large-cohort and statistically stringent approach clarifies this situation by showing damage to all four tracts, i.e., tracts connecting with anterior temporal cortices on the one side, and tracts connecting or passing nearby altered posterior temporal cortices, on the other side.

In lv-PPA, our findings are in line with numerous studies showing cortical damage to the temporal-parietal junction [e.g., (3, 10)]. However, early-stage extension to more anterior temporal cortices has not been evidenced in large-scale PPA studies but only in investigations which did not correct PET data for atrophy (13, 55) or which explored more advanced stages of lv-PPA (14, 15, 55). On the other hand, several studies found atrophy or hypometabolism extending to frontal regions and/or the right hemisphere (10, 13–16, 56). However, lv-PPA patients had longer disease duration in these studies. Besides, our findings in lv-PPA also show that metabolic alterations are more extensive than structural alterations, which is in line with clinical experience and with the results of one investigation conducted by Whitwell et al. (13). In addition, our findings show that tract alterations are not limited to the SLF and the ILF [e.g., (7)] but that they also involve the left IFOF. This finding is coherent with the fact that the IFOF runs nearby posterior temporal cortices and the temporal-parietal junction (48). The only study which has suggested alterations of the IFOF (16) was not based on the current diagnostic criteria of lv-PPA (1) given that it started before the publication of them. Furthermore, the patient population had a longer symptom duration than those of our study, and displayed diffuse cortical damage.

In nfv-PPA the thinning of left motor and premotor cortices is consistent with previous studies (17, 57). However, our results show that metabolic PET is required in early stages of the disease to reliably demonstrate damage to Broca's area which crucially contributes to syntactic processing [e.g., (58)] and phonological encoding [e.g., (59)], specifically altered in nfv-PPA. This differential sensitivity of cortical thickness and metabolic PET assessments has not been reliably demonstrated in previous studies given that the two investigations using PET and MRI either included small patient samples (6) or did not directly compare PET and cortical thickness data in early stages of nfv-PPA (20). In previous studies, alterations of Broca's area were observed using structural MRI (4, 17, 20, 60) but alterations in temporal or parietal regions were also detected. Only Spinelli et al. (12) found atrophy in Broca's area together with motor and premotor cortices which may indicate that atrophy of Broca's area appears after that of motor and premotor cortices. Regarding tract alterations in early stage nfv-PPA, we only found alterations in the UF while alterations of the SLF were reported in a previous study (7). This discrepancy might be explained by the very small number of nfv-PPA patients explored with DWI in our study or by different disease stages.

Regarding alterations across PPA variants, our findings indicate that sv-PPA and lv-PPA affect similar brain structures whereas nfv-PPA demonstrates a substantially distinct anatomical pattern. Thus, brain language networks appear to have differential vulnerability to degenerative processes as a function of the initial cortical lesion site. Networks in frontal regions dedicated to combinatorial operations of syntax and phonological assembly (49, 58, 59) are primarily affected by nfv-PPA whereas language networks in temporal regions dedicated to stored lexical and semantic information (50–53, 59, 61) are affected by both lv-PPA and sv-PPA. Hence, the classification of lv-PPA vs. sv-PPA can be difficult and lead to a non-negligible number of so-called “mixed” or “unclassifiable” PPA cases (62, 63). To address this issue and differentiate lv-PPA from sv-PPA peak alterations of cortical thinning and/or hypometabolism as well as the intensity of lexical vs. semantic impairment should be carefully evaluated.

It should be noted that anterior temporal anatomical/metabolic damage extension in lv-PPA and posterior temporal damage extension in sv-PPA might be interpreted as a clinical-imaging discrepancy. However, it has been shown that even early stage lv-PPA patients have subtle semantic disorders when tested with psycholinguistic paradigms (64), which is coherent with our imaging findings revealing the involvement of anterior portions of the temporal lobe contributing to semantics. Likewise, sv-PPA patients have, in addition to semantic breakdown, lexical disorders as revealed by psycholinguistic testing (64, 65), which is coherent with our imaging findings revealing the involvement of posterior portions of the temporal lobe implementing the mental lexicon.

Our study was devoted to group comparisons and not individual classification of patients. Nevertheless, our results may still bring interesting information on the respective usefulness of MRI and FDG-PET in the diagnosis of PPA variants. Our findings suggest different conclusions according to each PPA variant. In the sv-PPA variant, the ability of both modalities to detect alterations seems comparable which is supported by previous studies (2). In lv-PPA, PET imaging is overall more sensitive than structural MRI to detect alterations, a conclusion supported also by other studies (13, 15). In nfv-PPA, both modalities allowed to detect alterations but pointed to different anatomical areas, alterations of Broca's area being only detected with PET. Overall, our findings support those of Matias-Guiu et al. (66) reporting higher diagnostic accuracy for lv-PPA and nfv-PPA using FDG-PET imaging when compared to results of Sajjadi et al. (67) using structural MRI.

The main limitation of our study is the smaller sample size for the nfv-PPA variant (12 patients for T1-PET and 6 patients in the DWI cohort). This has led to reduced statistical power to detect alterations in this patient group. It is thus likely that additional alterations exist in nfv-PPA. This is particularly true for white matter alterations such as, for instance, tracts connecting frontal areas of atrophy. The unbalanced sample size between PPA variants may have led to detect only effects of large size in the nfv-PPA group while effects of smaller size could be detected for sv-PPA and lv-PPA. This should lead us to interpret with caution the very limited overlap that we observed between nfv-PPA and the other two variants.

The full-blown and statistically robust picture of brain alterations in early-stage PPA variants revealed by our findings enriches knowledge in the PPA field and potentially provides clues for future therapeutic strategies. The identification of the different cortical and sub-cortical structures specifically altered in PPA might open an avenue for trans-cranial brain stimulation approaches, such as Transcranial Magnetic Stimulation (TMS) or transcranial Direct Current Stimulation (tDCS), which interact with cortical regions and related language networks. In this vein, our detailed findings could indicate the appropriate entry into the damaged language system and thus provide valuable cortical target sites for TMS or tDCS trials in the different variants of PPA.

## CAPP study group

**Marc-Etienne Meyer**, MD, Ph.D., Department of Nuclear Medicine, Amiens University Hospital, coordination of PET imaging; **Pascal Bailly**, Department of Nuclear Medicine, Amiens University Hospital, coordination of PET imaging; **Hervé Deramond**, MD, Ph.D., Department of Neuroradiology Amiens University Hospital, coordination of MRI imaging; **Jean-Marc Constans**, MD, Ph.D., Department of Neuroradiology Amiens University Hospital, coordination of MRI imaging; **Candice Picard**, MD, Department of Neurology Amiens University Hospital, participant recruitment; **Martine Roussel**, Ph.D., Department of Neurology Amiens University Hospital, participant recruitment; **Charlotte Bigand**, speech therapist, Department of Neurology Amiens University Hospital, cognitive testing; **Pierre Vera**, MD, Ph.D., Department of Nuclear Medicine, Henri Becquerel Cancer Center and Rouen University Hospital, coordination of PET imaging; **Mathieu Chastan**, MD, Department of Nuclear Medicine, Henri Becquerel Cancer Center and Rouen University Hospital, coordination of PET imaging; **Emmanuel Gérardin**, MD, Ph.D., Department of Neuroradiology Rouen University Hospital, coordination of MRI imaging; **Carine Amossé**, speech therapist, Department of Neurology Rouen University Hospital, cognitive testing; **Sandrine Bioux**, neuropsychologist, Department of Neurology Rouen University Hospital, cognitive testing; **Léopoldine Deheinzelin**, neuropsychologist, Department of Neurology Rouen University Hospital, cognitive testing; **Evangéline Bliaux**, neuropsychologist, Department of Neurology Rouen University Hospital, cognitive testing; **Carole Girard**, neuropsychologist, Department of Neurology Rouen University Hospital, cognitive testing; **Dorothée Pouliquen**, neuropsychologist, Department of Neurology Rouen University Hospital, cognitive testing; **Pierre Payoux**, MD, Ph.D., Department of Nuclear Medicine Toulouse University Hospital, coordination of PET imaging; **Pierre Celsis**, MD, Ph.D., Department of Neuroradiology Toulouse University Hospital, coordination of MRI imaging; **Catherine Bezy**, speech therapist, Department of Neurology Toulouse University Hospital, cognitive testing; **Bérengère Pages**, MSc, neuropsychologist, Department of Neurology Toulouse University Hospital, cognitive testing; **Céline Gallazzini Crépin**, MD, Department of Nuclear Medicine Grenoble University Hospital, coordination of PET imaging; **Alexandre Krainik**, MD, Ph.D., Department of Neuroradiology Grenoble University Hospital, coordination of MRI imaging; **Stéphanie Maurice**, speech therapist, Department of Neurology Grenoble University Hospital, cognitive testing; **Marie-Pierre Brutti-Mairesse**, speech therapist, Department of Neurology Grenoble University Hospital, cognitive testing; **Annik Charnallet**, neuropsychologist, Ph.D., Department of Neurology Grenoble University Hospital, cognitive testing; **Sabrina Iannuzzi**, neuropsychologist, Department of Neurology Grenoble University Hospital, cognitive testing; **Alexandra Juphard**, neuropsychologist, Ph.D., Department of Neurology Grenoble University Hospital, cognitive testing; **Delphine Lassus-Sangosse**, neuropsychologist, Ph.D., Department of Neurology Grenoble University Hospital, cognitive testing; **Jacques Monteil**, MD, Ph.D., Department of Nuclear Medicine Limoges University Hospital, coordination of PET imaging; **Marie Paule Boncoeur**, MD, Department of Neuroradiology Limoges University Hospital, coordination of MRI imaging; **Leslie Cartz Piver**, MD, Department of Neurology, Limoges University Hospital, participant recruitment; **Marianne Chouly**, neuropsychologist, Department of Neurology Limoges University Hospital, cognitive testing; **Marie Nicol**, research assistant, Department of Neurology Limoges University Hospital, participant recruitment; **Alicia Sanchez**, MD, Department of Nuclear Medicine Saint Etienne University Hospital, coordination of PET imaging; **Fabrice-Guy Barral**, MD, Ph.D., Department of Neuroradiology Saint Etienne University Hospital, coordination of MRI imaging; **Olivier Couturier**, MD, Ph.D., Department of Nuclear Medicine Angers University Hospital, coordination of PET imaging; **Anne Pasco-Papon**, MD, Ph.D., Department of Neuroradiology Angers University Hospital, coordination of MRI imaging; **Valérie Chauviré**, MD, Department of Neurology Angers University Hospital, participant recruitment; **David Delafuys**, speech therapist, Department of Neurology Angers University Hospital, cognitive testing; **Didier Le Gall**, neuropsychologist, Ph.D., Department of Neurology Angers University Hospital, cognitive testing; **Delphine Boussard**, neuropsychologist, Department of Neurology Angers University Hospital, cognitive testing; **Nathalie Chanson**, neuropsychologist, Department of Neurology Angers University Hospital, cognitive testing; **Claude Hossein-Foucher**, MD, Department of Nuclear Medicine Lille University Hospital, coordination of PET imaging; **Christine Delmaire**, MD, Ph.D., Department of Neuroradiology Lille University Hospital, coordination of MRI imaging; **Vincent Deramecourt**, MD, Ph.D., Department of Neurology Lille University Hospital, participant recruitment; **Stéphanie Bombois**, MD, Ph.D., Department of Neurology Lille University Hospital, participant recruitment; **Yaohua Chen**, MD, Department of Neurology Lille University Hospital, participant recruitment; **Melanie Leroy**, research assistant, Department of Neurology Lille University Hospital, participant recruitment; **Nathalie Bout**, speech therapist, Department of Neurology Lille University Hospital, cognitive testing; **Justine Boutantin**, neuropsychologist, Department of Neurology Lille University Hospital, cognitive testing; **Florence Lejeune**, MD, Ph.D., Department of Nuclear Medicine Rennes University Hospital, coordination of PET imaging; **Jean Christophe Ferré**, MD, Ph.D., Department of Neuroradiology Rennes University Hospital, coordination of MRI imaging; **Catherine Merck**, neuropsychologist, Department of Neurology Rennes University Hospital, cognitive testing; **Amandine Pallardy**, MD, Department of Nuclear Medicine Nantes University Hospital, coordination of PET imaging; **François-Xavier Bertrand**, MD, Ph.D., Department of Neuroradiology Nantes University Hospital, coordination of MRI imaging; **Claire Boutoleau Bretonniére**, MD, Ph.D., Department of Neurology Nantes University Hospital, participant recruitment; **Delphine De Verbizier Lonjon**, MD, Department of Nuclear Medicine Montpellier University Hospital, Gui de Chauliac, coordination of PET imaging; **Alain Bonafé**, MD, Ph.D., Department of Neuroradiology Montpellier University Hospital, coordination of MRI imaging; **Nicolas Manjot de Champfleur**, MD, Ph.D., Department of Neuroradiology Montpellier University Hospital, coordination of MRI imaging; **Emmanuelle Le Bars**, Ph.D., Department of Neuroradiology Montpellier University Hospital, coordination of MRI imaging; **Caroline Grasselli**, MD, Department of Neurology Montpellier University Hospital, participant recruitment; **Audrey Gabelle**, MD, Ph.D., Department of Neurology Montpellier University Hospital, participant recruitment; **Sylvie Moritz**, Ph.D., neuropsychologist, Department of Neurology Montpellier University Hospital, cognitive testing; **Inna Digay-Cochet**, MD, Centre de Lutte contre le Cancer Georges Francois Leclerc, Dijon, coordination of PET imaging; **Laurent Vervueren**, MD, Angers University Hospital, coordination of PET imaging; **Marie-Odile Habert**, MD, Ph.D., Department of Nuclear Medicine, Pitié Salpêtrière Hospital Paris, coordination of PET imaging; **Aurélie Kas**, MD, Ph.D., Department of Nuclear Medicine, Pitié Salpêtrière Hospital Paris, coordination of PET imaging; **Stéphane Léhéricy**, MD, Ph.D., CENIR-ICM, coordination of MRI imaging; **Bruno Dubois**, MD, Ph.D., Department of Neurology Pitié Salpêtrière Hospital Paris, participant recruitment; **Richard Levy**, MD, Ph.D., Department of Neurology Pitié Salpêtrière Hospital Paris, participant recruitment; **Agnès Michon**, MD, Department of Neurology Pitié Salpêtrière Hospital Paris, participant recruitment; **Isabelle Leber**, MD, Ph.D., Department of Neurology Pitié Salpêtrière Hospital Paris, participant recruitment; **Sophie Ferrieux**, speech therapist, Department of Neurology Pitié Salpêtrière Hospital Paris, cognitive testing; **Marie Nogues**, speech therapist, Department of Neurology Pitié Salpêtrière Hospital Paris, cognitive testing; **Céline Arbizu**, speech therapist, Department of Neurology Pitié Salpêtrière Hospital Paris, cognitive testing; **Richard Gnassounou**, neuropsychologist, Department of Neurology Pitié Salpêtrière Hospital Paris, cognitive testing; **Dalila Samri**, neuropsychologist, Department of Neurology Pitié Salpêtrière Hospital Paris, cognitive testing; **Tiffany Landuré**, neuropsychologist, Department of Neurology Pitié Salpêtrière Hospital Paris, cognitive testing; **Marie Chupin**, engineer, Ph.D., CATI neuroimaging platform, ICM and Neurospin, Image acquisition and quality control; **Sonia Djobeir**, clinical research associate, CATI neuroimaging platform, ICM and Neurospin, Image acquisition and quality control; **Valérie Causse-Lemercier**, Department of Nuclear Medicine, Pitié Salpêtrière Hospital Paris, radiopharmacy; **Emmanuel Itti**, MD, Ph.D., Department of Nuclear Medicine, Paris Est Creteil University Hospital Henri Mondor; **Olivier Colliot**, Ph.D., ARAMIS team, ICM, Paris, coordination of MR imaging and image analysis; **Anne Bertrand**, MD, Ph.D., ARAMIS team, ICM, Paris, image analysis; **Alexandre Routier**, MSc, ARAMIS and FrontLab team, ICM, image analysis and data management; **Justine Mertz**, MSc, FrontLab team, ICM, data management; **Martina Sundqvist**, MSc, ARAMIS and FrontLab team, ICM, data management and image analysis; **Marc Teichmann**, MD, Ph.D., Department of Neurology Pitié Salpêtrière Hospital Paris.

## Author contributions

AR has full access to all the data of the study and takes responsibility for the integrity of the data and the accuracy of the data analyses. MT, OC, M-OH, and AR: Study concepts and study design. All authors: Acquisition, analysis or interpretation of data interpretation, manuscript drafting or manuscript revision for important intellectual content, approval of final version of submitted manuscript. AR: Statistical analysis. MT, M-OH, and OC: Study supervision.

### Conflict of interest statement

The authors declare that the research was conducted in the absence of any commercial or financial relationships that could be construed as a potential conflict of interest.

## Supplementary material

Supplementary Figure 1 Areas of significant hypometabolism in sv-PPA, lv-PPA and nfv-PPA, compared to healthy controls. The maps display p-values, corrected for multiple comparisons using cluster correction and a FWHM (Full Width at Half Maximum) at 8 mm.

Supplementary Table 1 Repartition of participants across centers for the T1-PET the T1-DWI cohorts.
