# Supplementary figures and images for: Structural, Microstructural, and Metabolic Alterations in Primary Progressive Aphasia Variants
Source: Front Neurol. 2018 Sep 18;9:766. doi: 10.3389/fneur.2018.00766 (PMC6153366; doi:10.3389/fneur.2018.00766)

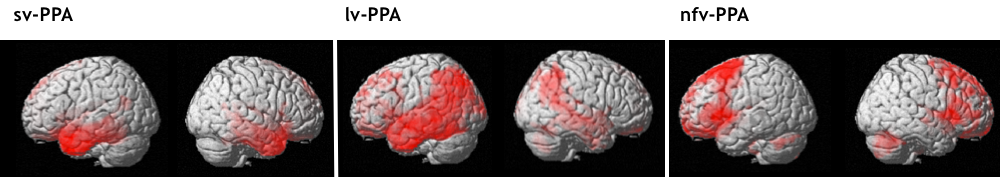

Supplement: Supplementary Figure 1 — Areas of significant hypometabolism in sv-PPA, lv-PPA and nfv-PPA, compared to healthy controls. The maps display p-values, corrected for multiple comparisons using cluster correction and a FWHM (Full Width at Half Maximum) at 8 mm. [file Image_1.TIFF]
