# Supplementary material for: Structural, Microstructural, and Metabolic Alterations in Primary Progressive Aphasia Variants
Source: Front Neurol. 2018 Sep 18;9:766. doi: 10.3389/fneur.2018.00766 (PMC6153366; doi:10.3389/fneur.2018.00766)
Supplement: Supplementary Table 1 — Repartition of participants across centers for the T1-PET the T1-DWI cohorts. [file Table_1.DOCX]

**Supplementary Table** Repartition of participants across centers for the T1-PET the T1-DWI cohorts.

**T1-PET Cohort**

|  | Amiens | Angers | Grenoble | Lille | Limoges | Montpellier | Nantes | Paris | Rennes | Rouen | Saint-Etienne | Toulouse |
| --- | --- | --- | --- | --- | --- | --- | --- | --- | --- | --- | --- | --- |
| nfv-PPA | 1 | 1 | 0 | 1 | 0 | 3 | 0 | 2 | 2 | 1 | 0 | 1 |
| lv-PPA | 1 | 2 | 3 | 3 | 0 | 1 | 2 | 8 | 0 | 1 | 2 | 3 |
| sv-PPA | 2 | 4 | 3 | 1 | 4 | 1 | 1 | 17 | 4 | 3 | 0 | 1 |
| Controls | 0 | 0 | 0 | 3 | 0 | 1 | 0 | 16 | 0 | 2 | 0 | 0 |
| Total | 4 | 7 | 6 | 8 | 4 | 6 | 3 | 43 | 6 | 7 | 2 | 5 |

**T1-DWI Cohort**

|  | Amiens | Grenoble | Lille | Limoges | Montpellier | Paris | Rennes | Rouen | Toulouse |
| --- | --- | --- | --- | --- | --- | --- | --- | --- | --- |
| nfv-PPA | 0 | 0 | 0 | 0 | 2 | 1 | 2 | 1 | 0 |
| lv-PPA | 0 | 3 | 2 | 0 | 1 | 8 | 1 | 1 | 3 |
| sv-PPA | 1 | 3 | 0 | 3 | 0 | 16 | 6 | 2 | 1 |
| Controls | 0 | 0 | 1 | 0 | 0 | 15 | 0 | 2 | 0 |
| Total | 1 | 6 | 3 | 3 | 3 | 41 | 9 | 6 | 4 |
